# Identification of novel senataxin mutations in Chinese patients with autosomal recessive cerebellar ataxias by targeted next-generation sequencing

**DOI:** 10.1186/s12883-016-0696-y

**Published:** 2016-09-20

**Authors:** Cong Lu, Yi-Cen Zheng, Yi Dong, Hong-Fu Li

**Affiliations:** 1 Department of Neurology and Research Center of Neurology in Second Affiliated Hospital, and the Collaborative Innovation Center for Brain Science, Zhejiang University School of Medicine, 88 Jiefang Road, Hangzhou, 310009 China; 2 Department of Neurology and Institute of Neurology, First Affiliated Hospital, Fujian Medical University, Fuzhou, China; 3 The High School Affiliated to Fudan University-WLSA Fudan Academy, Shanghai, China

**Keywords:** Autosomal recessive cerebellar ataxias, Targeted next-generation sequencing, Ataxia with oculomotor apraxia type 2, Senataxin

## Abstract

**Background:**

Autosomal recessive cerebellar ataxias (ARCA) are a group of neurodegenerative disorders characterized by early onset of gait impairment, disturbed limb coordination, dysarthria, and eye movement abnormalities, most likely due to the degeneration of cerebellum, brainstem, and spinal cord. Despite of the rarity, ARCA are both clinically and genetically heterogeneous. To date, more than 30 culprit genes have been identified in ARCA. Unraveling the specific causative mutation in cases with ARCA remains challenging so far.

**Methods:**

Three ARCA pedigrees of Chinese ancestry were recruited. Clinical features were evaluated and peripheral blood was collected after obtaining the written inform. Laboratory examinations, brain MRI, and EMG were performed for all the affected individuals. Genomic DNA was extracted, followed by the screening of GAA repeat expansion in *FXN* gene to exclude Friedreich’s ataxia. Targeted next-generation sequencing combining Sanger sequencing was performed in each proband of these families.

**Results:**

Compound heterozygous mutations, c.3190G > T (p.E1064X) and c.4883C > G (p.S1628X) of *senataxin (SETX)* gene were identified in one family with two affected cases. Both of the patients presented with early onset of unsteady walk, dysarthria, and diplopia. EMG test revealed decreased conduction velocity and evoked potential of both motor and sensory nerve. Moreover, elevated serum alpha-fetoprotein (AFP) and apparent cerebellar atrophy were observed. These features were typical features of ataxia with oculomotor apraxia type 2 (AOA2) and in line with the genetic results. However, no specific mutation was identified in the other two pedigrees.

**Conclusions:**

We identified novel compound heterozygous mutations of *SETX* in Chinese AOA2 pedigree, which broaden the mutation spectrum of *SETX*. To our knowledge, this is the first report concerning Chinese AOA2 cases with *SETX* mutations.

**Electronic supplementary material:**

The online version of this article (doi:10.1186/s12883-016-0696-y) contains supplementary material, which is available to authorized users.

## Background

Autosomal recessive cerebellar ataxias (ARCA) are a large group of neurodegenerative disorders characterized by autosomal recessive inheritance, cerebellar ataxia, and early onset of disease [1]. Clinically, ARCA are associated with diverse involvement of central and peripheral nervous systems, and with many systemic signs [2]. It comprises many subtypes, among which Friedreich’s ataxia (FA), ataxia telangiectasia (AT), and ataxia with oculomotor apraxia (AOA) are the most frequent [3]. The overall prevalence of ARCA is estimated to be about 5 to 6 per 100,000 [4]. Despite the rarity of ARCA, they are very heterogeneous in age at onset, manifestations, disease course, and progression. To date, more than 30 causative genes have been identified in ARCA [1, 2], which were therefore divided into many subtypes. Although many culprit genes have been identified, no genetic mutation or variance is detected in approximately half of the ARCA patients [5].

A simple and effective approach to detect the causative mutations in ARCA is warranted. Sanger sequencing is a classic and convenient method to screen genetic mutations or variants [6]. However, it is laborious and time-consuming. The high throughput whole exome or whole genome sequencing is too expensive to be widely applied [7]. The recently developed targeted next-generation sequencing (NGS) has shown distinct advantage in molecularly diagnosing inherited diseases, especially in neurologic disorders [6]. By now, it has been applied in inherited ataxia [8], amyotrophic lateral sclerosis (ALS) [9], Charcot-Marie-Tooth (CMT) [10], hereditary spastic paraplegia (HSP) [11], and so on. We have successfully used this technology to diagnose ALS and CMT [9, 10]. In this study, we collected three well defined ARCA families and screened the causative mutations using targeted NGS.

## Methods

### Subjects

**Fig. 1:**
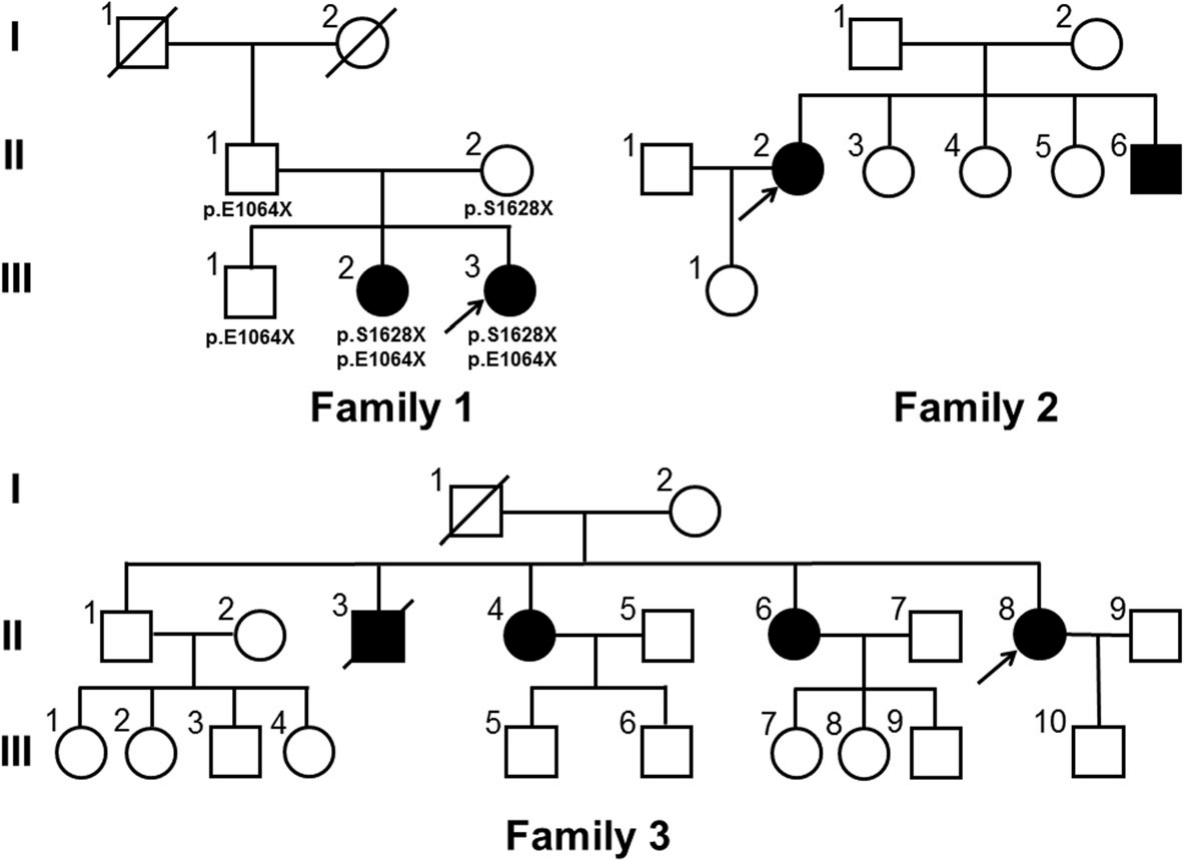
Pedigrees of 3 ARCA families. Squares indicate males; circles indicate females; the black symbols indicate affected individuals; diagonal lines across symbols indicate deceased individuals; arrows indicate the probands. Abbreviation: ARCA = autosomal recessive cerebellar ataxias

Three Chinese Han pedigrees with diagnosis of ARCA were enrolled (Fig. 1). The clinical presentations were evaluated by at least two senior neurologists. The laboratory tests, electromyography (EMG), and brain magnetic resonance imaging (MRI) scanning were carried out. Patients were assigned to an ARCA diagnosis if they presented cerebellar features (such as progressive limb/trunk ataxia, nystagmus, dysarthria, intentional tremor, and cerebellar atrophy on brain MRI) and autosomal recessive inheritance. A diagnosis of ARCA was made according to the Harding diagnostic criteria [12]. In addition, 500 healthy individuals of matched geographical ancestry were recruited as controls. Written informed consent was obtained from each participant and this study was approved by Ethic Committee of Second Affiliated Hospital, Zhejiang University School of Medicine.

### Targeted next-generation sequencing

Genomic DNA was extracted from 3 mL peripheral blood using QIAamp genomic DNA kits (Qiagen, Hilden, Germany). Before targeted NGS, all the probands were screened for GAA repeat expansion in frataxin (FXN) gene to exclude the possibility of FA [13]. Targeted capture, library preparation, and sequence amplification of 39 ARCA causative genes (seen in Additional file 1: Table S1) were then performed as previously described [9]. The deep sequencing was conducted on the Illumina HiSeq2000 platform (Genergy Biotechnology CoLtd, Shanghai, China).

### Sequence analysis

The sequenced reads were aligned to human reference genome (UCSC hg 19) (http://hgdownload.cse.ucsc.edu/) through Burrows-Wheeler Aligner [14]. We collected reads that were situated in the targeted regions for insertions, deletions and single nucleotide variants analysis. Genome Analysis Tool kit1.6 (GATK) tools were applied to call variants [15]. All annotation databases downloaded from the UCSC Genome Browser was used to annotate the variants [16]. It is hypothesized that the frequency of mutations causing ARCA should be low in general population. Therefore, variants with a minor allele frequency higher than 5 % in dbSNP database, 1000 Genomes Project, ExAc Browser, and Exome Sequencing Project were filtered out. Benign variants were identified and ruled out from the variant list by SIFT software (http://sift.jcvi.org/).

### Sanger sequencing

Sanger sequencing was performed on ABI 3730 DNA Sequencer to validate the filtered variants. For co-segregation analysis, all affected and unaffected familial members were screened to confirm the identified variants. Moreover, 500 unaffected healthy controls were sequenced for the detected mutations. The sequencing results were mapped to human reference genome published in Ensembl (http://www.ensembl.org/).

## Results

### Identification of variants in 39genes associated with ARCA by targeted NGS

Targeted NGS was performed in each proband (Family 1:III-3, Family 2:II-2, Family 3:II-8) of the three ARCA pedigrees. According to the high throughput of this approach, an average of 98.72 % targeted regions was covered with >50X. After single nucleotide variants, insertions and deletions calling, an average of 180.6 variants involving coding and noncoding regions was detected in each sample. Given that ARCA are a group of rare disorders, it is less possible that causative variants will be present in general population. Therefore, variants formerly published in dbSNP database, 1000 Genomes Project, ExAc Browser, and Exome Sequencing Project were excluded. After the initial filtration, the candidate variants were reduced to 7.3 per proband. The damage variants were then prioritized by SIFT software. After this filtering, the candidate variants were further decreased to 6.6 per subject (Additional file 2: Table S2).

### Two novel mutations in SETX confirmed by Sanger sequencing

**Fig. 2:**
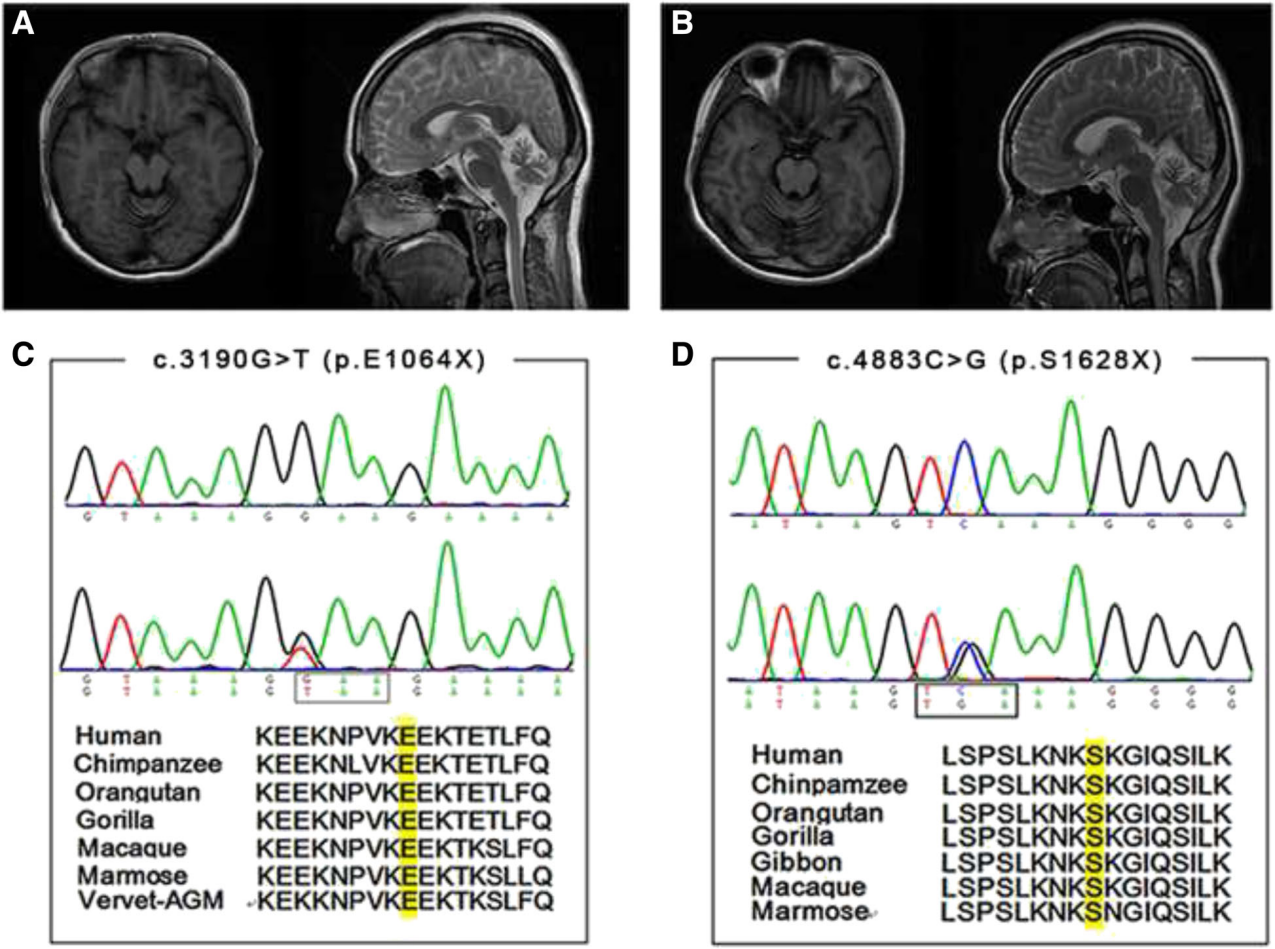
Brain magnetic resonance imaging of the proband III-3 (**a**) and the patient III-2(**b**) in Family 1. *Left*: axial T1-weighted image showing atrophy of the cerebellar vermis. *Right*: mid-line sagittal T2-weighted image showing cerebellar atrophy, particularly evident in the vermis and hemispheres, with enlargement of the fourth ventricle. Chromatogram and conservation of p.E1064X mutation (**c**) and p.S1628X mutation (**d**) within *SETX*. The upper panel depicts the reference sequence. The lower panel represents heterozygous mutated sequence

As the ARCA was inherited in an autosomal recessive manner, we were then focused on homozygous and compound heterozygous mutations. After verification by Sanger sequencing, we identified compound heterozygous *SETX* mutations, c.3190G > T (p.E1064X) and c.4883C > G (p.S1628X), in the proband (III-3) of **Family 1** (Fig. 2c,d). Both of these two mutations were nonsense mutations and located in exon 10, a relatively conserved medium region of *SETX*.A further validation revealed that the proband’s sister (III-2) carried p.S1628X and p.E1064X mutation too, while her mother carried heterozygous p.S1628X and her father carried heterozygous p.E1064X. These suggested that the identified mutations co-segregated with the disease in **Family 1**. In addition, these mutations were absent in 500 matched controls. However, no mutation was found in the other two probands.

### The clinical presentations of patients carrying *SETX* mutations

The detailed clinical characteristics of four ARCA patients were summarized in Table 1. The proband (III-3) in **Family 1** was a 23-year-old female who had 7 years of progressive gait difficulty. She had normal delivery and postnatal period. Her developmental milestones were unremarkable until the age of 16, when she exhibited unstable gait and slightly slurred speech. She felt like walking on cotton and fell to the ground sometime. EMG was not performed at that time. Two years after the onset, she recognized that her speech disturbance was worsened and she could not write neatly and use chopsticks stably. In the following one year, she presented with dysarthria and double vision. She noticed that her stagger was aggravated in the dark. She also complained that she often forgot the trifle things of life. Her swallowing was essentially normal with rare bucking after drinking. When she sought her medical attention in our hospital at age of 22, she exhibited severe gait unsteadiness, dysarthria, and slurred speech. Neurological examinations revealed horizontal nystagmus but normal ocular movements. No amyotrophy of muscle was noticed. The muscle strength and tension were normal. There was no abnormity of sense in her limbs and trunk. The deep tendon reflexes were decreased in her lower limbs, and Babinski’s sign was bilaterally positive. Finger-nose test and heel-knee-shin test were abnormal. Romberg’s sign was positive. Mini Mental State Examination (MMSE) revealed a score of 29/30, suggesting that her recognition was not impaired. Laboratory findings revealed normal albumin, ferritin, Vitamin B12, ceruloplasmin, cholesterol, and IgG. However, her serum a-fetoprotein (AFP) was elevated (40.3, normal <20 ng/ml) and the serum level of low density lipoprotein (LDL) was reduced (1.79, normal 2.1 ~ 3.1nmol/L). Lumbar puncture revealed uninformative findings. EMG test revealed decreased conduction velocity and evoked potential of both motor and sensory nerve. Brain MRI demonstrated atrophy of cerebellar predominantly in vermis and hemispheres (Fig. 2a, b).

**Table 1:** Clinical features of patients with autosomal recessive cerebellar

	III-2 (Family 1)	III-3 (Family 1)	II-2 (Family 2)	II-8 (Family 3)
Gender	F	F	F	F
Age at onset (years)	20	16	30	25
Disease duration (years)	5	7	20	19
Initial symptom	GU	GU/SS	GU	GU/SS/T
Ataxia	TL	L	L	TL
Nystagmus	H	V	_	H
Slow saccade	_	+	_	_
Diplopia	+	+	_	_
Dysarthria	+	+	+	+
Tremor	TL	L	_	TL
Pes cavus	+	+	_	_
Tendon reflexes	absent	decreased	decreased	left brisk
Babinski sign	right positive	bilateral positive	negative	NA
AFP (<20 ng/ml)	81.1	40.3	9.74	NA
LDL (2.1 ~ 3.1 ng/mL)	1.68	1.79	4.16	NA
cholesterol (3.1–6 nmol/L)	3.90	3.45	7.84	NA
triglyceride (<1.69 nmol/L)	0.46	0.71	2.76	NA
MMSE (30)	28	29	NA	NA
EMG	SMN	SMN	NA	NA
Brain MRI	CA	CA	CA	CA
EEG	borderline	normal	NA	normal
Other features	hand deformity	hand deformity	diabetes; macula lutea impairment	myoclonic jerks; involuntary movement

*CA* cerebellar atrophy, *F* female, *GU* gait unsteadiness, *H* horizontal, *HV* horizontal and vertical, *L* limbs, *NA* non-available, *T* termor, *TL* trunk and limbs, *SMN* sensorimotor neuropathy, *SS* screening speech, + positive, _ negative

The proband’s 25-year-old sister (III-2) had similar symptoms to her. At the age of 20, she began to feel dizziness and noticed gait difficulty. One year later, her walk unsteadiness was worsened, especially in the dark. In the following half one year, she gradually exhibited slurred speech and diplopia. Swallowing was not impaired. Two years after the onset, she developed tremor of both hands. When she was admitted in our hospital, she presented with noticeable ataxia, poem-like language, and intention tremor. She could not walk without the help of others. On cranial nerve examinations, she showed horizontal and vertical nystagmus in both eyes. Noticeable amyotrophy was observed in her lower limbs. However, the muscle strength, tension, and sense were basically normal. Deep tendon reflexes were disappeared in lower limbs and decreased in the upper limbs. Right Babinski’s sign was positive. Finger-nose test and heel-knee-shin test were clumsy, with decomposition and dysmetria on both sides. MMSE test was unremarkable, with a score of 28/30. Blood inspections revealed an increase of serum AFP (81.8, normal <20 ng/ml) and a decreased LDL (1.68, normal 2.1 ~ 3.1nmol/L). Other laboratory results were uninformative and cerebrospinal fluid examinations were normal. EMG test revealed reduced conduction velocity and evoked potential of motor nerve. The velocity of bilateral sural nerve and peroneus superficial nerve could not be detected. The evoked potential of sense nerves was decreased. Brain MRI showed apparent cerebellar atrophy.

The proband’s brother (III-1) was 28 years old at present. He did not complain about any gait problem, slurred speech, or diplopia. His serum AFP was within normal range. Brain MRI was unremarkable. Genetic investigations revealed a heterozygous p.E1064X mutation.

## Discussion

In this study, we collected three ARCA pedigrees of Chinese ancestry and performed targeted next-generation sequencing in each proband of these families. After verification by Sanger sequencing, we identified compound heterozygous mutations (p.S1628X/p.E1064X) within *SETX* in one family, in which there were two affected cases and one healthy sibling. Both of the two patients presented with unsteady walk, dysarthria, and diplopia. EMG test revealed peripheral nerve damage. Moreover, elevated serum AFP and apparent cerebellar atrophy were observed in these two cases. Taken together, these were in line with the features of AOA2, which was characterized by juvenile-onset cerebellar ataxia, oculomotor apraxia, sensorimotor neuropathy, marked cerebellar atrophy, and elevated serum AFP [17]. The prevalence of AOA2 was low worldwide and rare in Asian ethnicity. Of note, this disease has not been documented in Chinese Han population so far. To our knowledge, this is the first report of Chinese AOA2 cases.

To date, near 100 mutations within *SETX* have been identified in AOA2 cases (http://www.hgmd.cf.ac.uk/). Among these mutations, there is a maximum ratio in exon 10 of *SETX.* In this study, the identified p.S1628X and p.E1064Xmutations were also located in the exon 10. Due to premature of stop codon, these two nonsense mutations brought about the loss of senataxin’s essential role in DNA repair and RNA processing [18]. In addition, these two mutation were predicted to be disease causing by the SIFT. Although we did not perform functional experiments in this study, these two mutations were regarded as pathogenic mutations for the AOA2 in this family, according to the American College of Medical Genetics and Genomics (ACMG) standards and guidelines [19]. However, further functional investigations were required to elucidate the pathogenicity of the identified novel mutations in the future.

Oculomotor apraxia is a specific feature of AOA2, but not all the AOA2 cases exhibited abnormal ocular movements. Approximately half of AOA2 cases were reported to have oculomotor apraxia [17, 20, 21]. In this study, both the cases in Family 1 had diplopia, but their ocular movements were basically normal. This suggested that oculomotor apraxia might not be the essential factor for the diagnosis of AOA2. Alternatively, the cases have not developed oculomotor apraxia yet because of the early stage of the disease. A subsequent follow-up is required in the future. In addition, the phenotype of these two cases was not identical, albeit they carried the same *SETX* mutations. The older sister (III-2) had a later onset of ataxia but a more severe disability than that in the proband (III-3). She tended to have a more rapid progression. Since the sister had higher serum AFP, we speculated that AFP might have important effect on the pathogenesis of AOA2. Certainly, further research about the role of elevated serum AFP in neurologic diseases is warranted in large sample. Additionally, the disease course and prognosis of these two cases need further follow up.

Due to the rarity of AOA2, accurate diagnosis at early stage of the disease remains challenging in clinical practice. For these cases with early onset of ataxia, oculomotor impairment, and recessive history, it is necessary to inspect serum AFP and make EMG test. If the serum AFP is elevated, a diagnosis of AT or AOA2 should be considered. If EMG further reveals peripheral nerve damage, a suspected diagnosis of AOA2 can be made and genetic screening of SETX mutations is needed. Of course, it is still challenging to decide which gene should be screened solely depending on the cases’ clinical features. On one hand, the phenotypes of ARCA were heterogeneous. On the other hand, the manifestations of patients were varying at different stage of the disease. For this reason, targeted NGS enables us to capture and sequence multiple genomic regions of interest. It is promising that this technique may become a routine approach to molecularly diagnose inherited diseases in the future. However, the difficulty in detecting nucleotide repeat expansions is the limitation of this approach and may result in the loss of some valuable information and artificial bias for assessment.

## Conclusions

In summary, we identified novel compound heterozygous mutations in SETX by targeted next-generation sequencing, which broaden the spectrum of SETX mutations. Although the role of AFP in the pathogenesis of AOA2 remains unclear, it seems to be a useful biomarker in the diagnosis of AOA2. In the other two pedigrees, we did not identify any causative mutations. It is possible that there may be some newly causative genes unselected in our targeted panel. Alternatively, they may be caused by novel nucleotide repeat expansions which were not detected by routine screening. These two pedigrees need further genetic investigations in the future. With the improvements of NGS technology and the bioinformatic analysis approach, it is believed that the individuals with hereditary neurologic disorder may eventually have a molecular diagnosis through NGS approach.

## Additional files

Additional file 1: List of genes responsible for ARCA. (DOCX 28 kb) Additional file 2: Summary of gene variants detected by targeted sequencing. (DOCX 29 kb)

## Abbreviations

AFP: Alpha-fetoprotein
AOA2: Ataxia with oculomotor apraxia type 2
ARCA: Autosomal recessive cerebellar ataxias
SETX: Senataxin gene
