# Supplementary material for: Identification of novel senataxin mutations in Chinese patients with autosomal recessive cerebellar ataxias by targeted next-generation sequencing
Source: BMC Neurol. 2016 Sep 20;16:179. doi: 10.1186/s12883-016-0696-y (PMC5029030; doi:10.1186/s12883-016-0696-y)
Supplement: Additional file 1: — List of genes responsible for ARCA. (DOCX 28 kb) [file 12883_2016_696_MOESM1_ESM.docx]

**Supplementary Table 1.** List of genes responsible for ARCA

| **Number** | **Disease** | **Gene symbol** | **Chromosomal position** |
| --- | --- | --- | --- |
| 1 | AOA1 | APTX | 9p13.3 |
| 2 | AOA2 | SETX | 9q34.13 |
| 3 | AOA3 | PIK3R5 | 17p13.1 |
| 4 | SCAN1 | TDP1 | 14q32.11 |
| 5 | SCAR5 | ZNF592 | 15q25.3 |
| 6 | SCAR7 | TPP1 | 11p15 |
| 7 | SCAR8 | SYNE1 | 6q25 |
| 8 | SCAR9 | ADCK3 | 1q42.13 |
| 9 | SCAR10 | ANO10 | 3p22.1 |
| 10 | SCAR11 | SYT14 | 1q32.2 |
| 11 | SCAR12 | WWOX | 16q23 |
| 12 | SCAR13 | GRM1 | 6q24 |
| 13 | SCAR14 | SPTBN2 | 11q13 |
| 14 | SCAR15 | KIAA0226 | 3q29 |
| 15 | SCAR16 | STUB1 | 16p13.3 |
| 16 | AT | ATM | 11q22-q23 |
| 17 | CTX | CYP27A1 | 2q35 |
| 18 | PACA | PTF1A | 10p12.2 |
| 19 | NA | AMACR | 5p13 |
| 20 | Refsum disease | PHYH | 10p13 |
| 21 | Refsum disease | PEX7 | 6q23.3 |
| 22 | BNHS | PNPLA6 | 19p13.2 |
| 23 | MSS | SIL1 | 5q31 |
| 24 | BVVLS1 | SLC52A3 | 20p13 |
| 25 | BVVSL2 | SLC52A2 | 8q24.3 |
| 26 | AXED | TTPA | 8q12.3 |
| 27 | WFS | WFS1 | 4p16.1 |
| 28 | PHARC | ABHD12 | 20p11.21 |
| 29 | ICRD | ACO2 | 22q13.2 |
| 30 | ATCAY | ATCAY | 19p13.3 |
| 31 | CAMRQ1 | VLDLR | 9p24 |
| 32 | CAMRQ2 | WDR81 | 17p13.3 |
| 33 | CAMRQ3 | CA8 | 8q12.1 |
| 34 | CAMRQ4 | ATP8A2 | 13q12 |
| 35 | LKPAT | CLCN2 | 3q27.1 |
| 36 | AXPC1 | FLVCR1 | 1q32.3 |
| 37 | SESAME | KCNJ10 | 1q23.2 |
| 38 | PTBHS | LAMA1 | 18p11.3 |
| 39 | SANDO | POLG | 15q25 |
