# Supplementary material for: Identification of novel senataxin mutations in Chinese patients with autosomal recessive cerebellar ataxias by targeted next-generation sequencing
Source: BMC Neurol. 2016 Sep 20;16:179. doi: 10.1186/s12883-016-0696-y (PMC5029030; doi:10.1186/s12883-016-0696-y)
Supplement: Additional file 2: — Summary of gene variants detected by targeted sequencing. (DOCX 29 kb) [file 12883_2016_696_MOESM2_ESM.docx]

**Supplementary Table 2.** Summary of gene variants detected by targeted sequencing.

III-3 (Family 1) II-2 (Family 2) II-8 (Family 3)

Total number of variants 195 193 154

Number of variants not in databases* 7 10 5

Number of variants filtered by SIFT 6 9 5

*databases include dbSNP, 1000 Genomes Project, ExAc, and Exome Sequencing Project
